# Gut Microbiota and Sinusoidal Vasoregulation in MASLD: A Portal Perspective

**DOI:** 10.3390/metabo14060324

**Published:** 2024-06-07

**Authors:** Gyorgy Baffy, Piero Portincasa

**Affiliations:** 1 Section of Gastroenterology, Department of Medicine, VA Boston Healthcare System, Boston, MA 02130, USA; 2 Department of Medicine, Brigham and Women’s Hospital, Harvard Medical School, Boston, MA 02115, USA; 3 Division of Internal Medicine, Department of Precision and Regenerative Medicine, University ‘Aldo Moro’ Medical School, 70121 Bari, Italy; piero.portincasa@uniba.it

**Keywords:** intrahepatic vascular resistance, portal hypertension, endohepatology, EUS-guided measurement of portal pressure gradient

## Abstract

Metabolic dysfunction-associated steatotic liver disease (MASLD) is a common condition with heterogeneous outcomes difficult to predict at the individual level. Feared complications of advanced MASLD are linked to clinically significant portal hypertension and are initiated by functional and mechanical changes in the unique sinusoidal capillary network of the liver. Early sinusoidal vasoregulatory changes in MASLD lead to increased intrahepatic vascular resistance and represent the beginning of portal hypertension. In addition, the composition and function of gut microbiota in MASLD are distinctly different from the healthy state, and multiple lines of evidence demonstrate the association of dysbiosis with these vasoregulatory changes. The gut microbiota is involved in the biotransformation of nutrients, production of de novo metabolites, release of microbial structural components, and impairment of the intestinal barrier with impact on innate immune responses, metabolism, inflammation, fibrosis, and vasoregulation in the liver and beyond. The gut–liver axis is a conceptual framework in which portal circulation is the primary connection between gut microbiota and the liver. Accordingly, biochemical and hemodynamic attributes of portal circulation may hold the key to better understanding and predicting disease progression in MASLD. However, many specific details remain hidden due to limited access to the portal circulation, indicating a major unmet need for the development of innovative diagnostic tools to analyze portal metabolites and explore their effect on health and disease. We also need to safely and reliably monitor portal hemodynamics with the goal of providing preventive and curative interventions in all stages of MASLD. Here, we review recent advances that link portal metabolomics to altered sinusoidal vasoregulation and may allow for new insights into the development of portal hypertension in MASLD.

## 1. Introduction

Metabolic dysfunction-associated steatotic liver disease (MASLD, formerly known as nonalcoholic fatty liver disease or NAFLD [1]) is estimated to affect up to 30% of the general adult population, representing the most prevalent liver disorder of our time [2]. MASLD is an example of a complex disease: it has a pathogenesis epitomized by insulin resistance, and it is driven by genetic predisposition and environmental factors. MASLD also clusters with other metabolic disorders such as obesity, type 2 diabetes, and metabolic syndrome, and its clinical outcomes are difficult to predict at the individual level [3,4,5]. A key histological feature of MASLD is hepatic triglyceride accumulation (steatosis), often associated with liver inflammation and cellular injury described as metabolic dysfunction-associated steatohepatitis (MASH, formerly known as nonalcoholic steatohepatitis or NASH), variable degree of fibrosis, and a potential to progress into cirrhosis [6].

Life-threatening complications of cirrhosis are mostly linked to portal hypertension, initiated by hemodynamic changes within the unique sinusoidal capillary network of the liver. The highly vascular liver receives a quarter of the cardiac output via the portal vein and the hepatic artery, which merge to form the low-pressure, low-flow sinusoids before draining to the hepatic vein [7]. Blood pressure differences between the portal vein and hepatic vein reflect the portosystemic pressure gradient (PPG), which does not exceed 5 mm Hg in healthy liver [8]. Due to difficult access to the portal vein, PPG is usually estimated by the hepatic venous pressure gradient (HVPG), which is the difference between the wedged hepatic venous pressure (WHVP) and the free hepatic venous pressure (FHVP), measured at the respective wedged and free-floating positions of a retrograde inserted hepatic vein catheter [8]. Subclinical portal hypertension is defined as HVPG > 5 mm Hg and <10 mm Hg, while major adverse clinical events have been associated with clinically significant portal hypertension (CSPH), defined as HVPG ≥ 10 mm Hg [9,10].

Portal hypertension in cirrhosis is initiated by increased intrahepatic vascular resistance, followed by splanchnic and systemic vasoregulatory changes, and by the formation of portosystemic collaterals upon further progression [8]. Experimental data and human observations indicate that intrahepatic vascular resistance begins to rise early in MASLD [11,12,13], although CSPH has rarely been found in the absence of cirrhosis, and the pathophysiology and clinical importance of these initial vasoregulatory changes is incompletely understood [14,15]. There is evidence that both functional and mechanical impediments of hepatic microcirculation can contribute to increased intrahepatic vascular resistance from the beginning of MASLD [16,17]. All major liver cell types, including parenchymal hepatocytes, liver sinusoidal endothelial cells (LSECs), hepatic stellate cells (HSCs), and Kupffer cells, have been implicated in the development of subclinical portal hypertension via cellular dysfunction and a web of cell–cell interactions [18,19]. It is also evident that sinusoidal vasoregulation in MASLD involves substances derived from the diet, the host, and the gut microbiota and delivered to the liver via the portal vein as part of an inter-organ dialog termed gut–liver axis [20,21].

From the earliest stages of MASLD, the composition and function of gut microbiota show distinct changes that have been associated with altered microbial metabolism and impairment of the intestinal barrier [22,23,24]. Under these conditions, exposure to changes in the amount and composition of portal-derived substances may unfavorably affect the liver and contribute to the progression of MASLD [25,26,27]. Recent efforts have focused on predicting the risk of disease progression from metagenomic signatures linked to clinical and metabolomic data [28,29,30]. There are many microbiota-derived metabolites and structural components that could potentially contribute to liver disease, while only a fraction of these have been characterized [31]. Biomarker research exploring the association between the gut microbial metagenome and different metabolic phenotypes of MASLD has been mostly based on the analysis of peripheral blood samples, which may not accurately reflect changes that occur in the portal circulation. Recently developed methods such as endoscopic ultrasound (EUS)-guided sampling of the portal and hepatic vein may allow easier and safer access to this hidden vascular territory and allow better insights into the pathogenesis, predict outcomes, and monitor responses to therapeutic interventions in MASLD and other liver diseases [32,33,34].

This narrative review will discuss recent advances that link portal metabolomics to altered sinusoidal vasoregulation and may serve as an impetus to gain new insights into the development of portal hypertension in MASLD.

## 2. Gut Microbiota and Dysbiosis in MASLD

Gut microbiota consists of more than a thousand different species and about 100 trillion microbial cells [35,36]. This large consortium of microorganisms includes fungi, viruses, and archea, while two bacterial phyla (*Bacteroides* and *Firmicutes*, recently reclassified as *Bacillota*) account for more than 90% of the human gut microbiota [37]. The altered composition and function of the commensal microbial community, termed dysbiosis, has been associated with MASLD severity. Thus, diminished microbial diversity is present in the early stages of MASLD, and the changes become more prominent with disease progression [38,39]. Decreased richness of the gut microbiota among participants with MASLD has recently been documented in the prospective population-based Rotterdam Study [40]. In another work, an analysis of the stool metagenome has identified distinct features in patients with biopsy-proven MASLD and helped distinguish mild-to-moderate from advanced fibrosis [28]. In a small cohort of patients with biopsy-proven MASLD, complex analysis of clinical, metabolomic, and metagenomic parameters has resulted in accurate prediction of progression from steatosis to steatohepatitis [41]. Similarly, a combination of metagenomics and metabolomics has identified parameters specific for steatosis and fibrosis independent of metabolic risk factors in a cohort of patients with MASLD and type 2 diabetes [27]. However, metabolic profiling in these studies was based on the analysis of peripheral blood samples, and no information was obtained on the portal metabolome.

**Figure 1:**
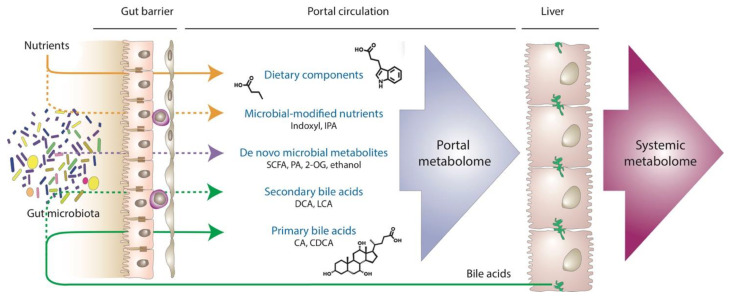
**The portal metabolome.** Origin of major classes of known metabolites absorbed through the gut epithelial and endothelial barrier and delivered to the liver via the portal circulation is schematically illustrated. Solid lines represent metabolites unmodified by the gut microbiota, dashed lines indicate metabolites synthesized de novo (e.g., SCFA [48], 2-OG [45], ethanol [43], or PA [47]) and modified (e.g., secondary bile acids such as DCA and LCA [49,50] or tryptophane derivatives such as indoxyl or IPA [51,52,53]) by the gut microbiota. Composition of the portal and systemic metabolomes may be vastly different due to the extraction, modification, and de novo synthesis of biomolecules in the liver (first pass effect), indicating the need for direct exploration of the portal metabolome. 2-OG, 2-oleoylglycerol; CA, cholic acid; CDCA, chenodeoxycholic acid; DCA, deoxycholic acid; IPA, indole-3-propionate; LCA, lithocholic acid; PA, phenylacetate; SCFA, short-chain fatty acid.

A large number of gut microbiota-derived products reach the liver through the gut barrier assembly and via the portal circulation [42]. These substances include host products that are modified by gut microbiota (e.g., secondary bile acids), dietary components metabolized by gut microbiota (e.g., short-chain fatty acids or SCFAs, amino acid metabolites such as tryptophan derivatives), or de novo gut microbial metabolites (e.g., phenylacetate, endogenous ethanol) and structural components (e.g., lipopolysaccharide or lipoteichoic acid) [43,44,45,46,47] (Figure 1). Some of these biomolecules have been implicated in the development and progression of MASLD, while others may have biological activities that remain unknown [31]. While metabolomic analysis of the portal blood pertains to small molecule metabolites (<1500 Da) produced by either the host or the gut microbial community, larger microbiota-derived biomolecules may significantly affect the pathogenesis of MASLD through evoking innate immune responses as discussed below.

Many physiological functions of the human body depend on commensal microorganisms, but gut microbiota-derived substances are not necessarily meant to reach the portal circulation [54]. The intestinal barrier, which consists of mucin, microbes, intestinal secretions and propulsion, and epithelial (mucosal), immunological, and endothelial (vascular) components [42], protects from potentially harmful substances, including microbial metabolites, structural components, or viable microorganisms entering the host circulation [55]. In addition, dysbiosis may impair microbial secretion of antibacterial substances that prevent colonization of pathogenic bacteria that would lead to undesirable responses in the host liver and beyond [56]. Multiple lines of experimental evidence support this view. Thus, fecal microbiota transplantation (FMT) to restore healthy microbiota has been shown to prevent diet-induced hepatic steatosis and fibrosis in a mouse model of experimental MASLD [57]. In addition, diet-induced steatohepatitis does not develop in germ-free mice [58] or in mice treated to prevent leakage of the intestinal barrier [26].

Short-chain fatty acids (SCFAs; primarily acetate, propionate, and butyrate) are derived from microbial fermentation of undigestible dietary fibers and other carbohydrates (i.e., microbiota-accessible carbohydrates), utilized in hepatic energy metabolism (e.g., gluconeogenesis or de novo lipogenesis) and biochemical signaling pathways (e.g., nutrient sensing or immune regulation) [56,59,60]. Since most SCFAs are cleared by the hepatic first-pass effect, the concentration of SCFAs in the portal vein is significantly higher compared to the systemic circulation [48]. In patients with cirrhosis, portal blood levels of SCFAs (mainly butyrate) show inverse relationships with scores such as the model for end-stage liver disease (MELD) and with decompensation events such as ascites and hepatic encephalopathy [61]. No similar data are available on the SCFA composition of portal blood in MASLD. In one of the few studies analyzing portal blood samples in MASLD, phospholipid profiling was performed in a cohort of 46 women undergoing bariatric surgery in comparison with lipid levels in peripheral blood and adipose tissue depots [62]. The presence of MASH in this cohort correlated with increased levels of phosphoglycerols (PG) and phosphoethanolamines (PE), which are components of bacterial membranes, suggesting that increased amounts of these potentially toxic lipids are released from gut microbiota to the portal circulation [62].

Bile acids are metabolites of cholesterol and represent an extensively studied group of bioactive substances in portal blood. Primary bile acids are synthesized from cholesterol in hepatocytes and secreted with bile into the gut lumen, where they almost entirely get reabsorbed and return to the liver via the portal vein in their primary form or after being modified into secondary bile acids by the gut microbiota [49]. Primary bile acids, such as cholic acid and chenodeoxycholic acid, preferentially target the farnesoid X receptor (FXR), while secondary bile acids, such as deoxycholic acid and lithocholic acid, stimulate the G protein-coupled bile acid receptor 1 or GPBAR1 (also known as TGR5). These bile acid-activated pathways substantially overlap, and the balance between FXR and GPBAR1 signaling depends on gut microbial action, which determines the ratio of primary and secondary bile acids through a series of complex biochemical transformations involving the continuous recycling of the bile acid pool within the enterohepatic circulation [49,50]. Changes in the composition of circulating bile acids seen in patients with MASLD have been associated with impaired FXR signaling and coincide with the presence of liver cell ballooning and fibrosis [63]. Bidirectionality of the gut–liver axis has been demonstrated by experiments in which genetic ablation of *Fxr* and *Gpbar1* in mice resulted in dysbiosis and increased gut permeability along with dysregulated bile acid synthesis [64].

## 3. Vasoregulatory Effects of Microbiota-Derived Substances in MASLD

**Figure 2:**
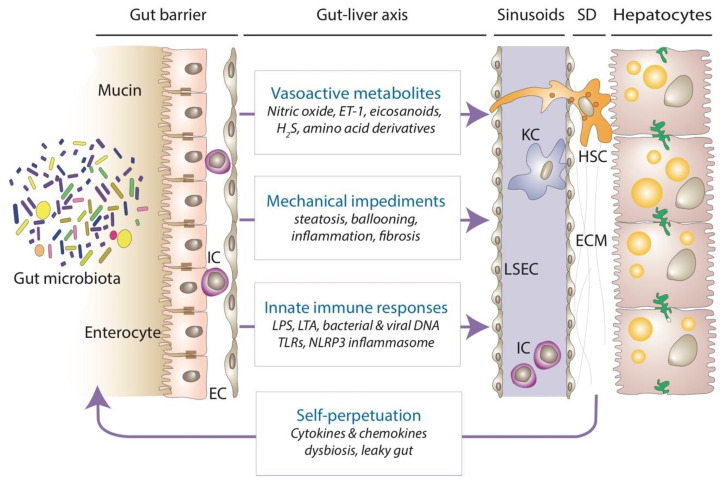
**Sinusoidal vasoregulation and gut–liver interactions in MASLD.** Major mechanisms affecting sinusoidal vasoregulation and intrahepatic vascular resistance by altered gut microbiota composition and function (dysbiosis) and impaired intestinal barrier (‘leaky gut’) in MASLD are schematically illustrated. Gut microbiota may adversely impact liver hemodynamics by modulating availability and function of directly acting vasoregulatory substances such as nitric oxide [67], endothelin-1 [67,68], eicosanoids [67,69], hydrogen sulfide [70,71], or amino acid derivatives [72,73,74]; by aggravating mechanical barriers to sinusoidal flow via space-occupying features of MASLD such as steatosis, ballooning, or interstitial edema [68,75]; or by stimulating pathogen recognition pathways via TLRs and the NLRP3 inflammasome by microbial associated molecular patterns such as LPS, LTA, bacterial and viral DNA [76,77,78,79] and promoting cell–cell interactions that disrupt sinusoidal vasoregulation. In addition, gut–liver interactions may induce self-perpetuating cycles by worsening dysbiosis and further weakening the intestinal barrier [23,76]. EC, endothelial cell; ECM, extracellular matrix; ET-1, endothelin-1; H_2_S, hydrogen sulfide; HSC, hepatic stellate cell; IC, immune cells; KC, Kupffer cell; LPS, lipopolysaccharide; LSEC, liver sinusoidal endothelial cell; LTA, lipoteichoic acid; MASLD, metabolic dysfunction-associated steatotic liver disease; NLRP3, nucleotide-binding domain, leucine-rich–containing family, pyrin domain-containing-3; SD, space of Disse.

While the gut–liver axis has been implicated in the pathogenesis of MASLD from the earliest stages of the disease, there is relatively little known about the impact of gut microbiota on sinusoidal circulation and the development of portal hypertension. In general, gut microbiota may affect hepatic vasoregulation by at least three different mechanisms: (1) producing or modifying metabolites that have vasoactive properties or represent precursors of vasoactive mediators directly acting on the liver vasculature; (2) augmenting the mechanical impediments of sinusoidal circulation by promoting steatosis, steatohepatitis, and fibrosis; and (3) stimulating the innate immune system and initiating adverse cell–cell interactions in the liver exposed to microbial components (Figure 2). There is also a mechanism of self-amplification due to the bidirectional relationship between the liver and the gut microbiota. The insults that drive chronic liver disease, such as sustained caloric excess fueling metabolic dysfunction in MASLD, may also have an adverse effect on gut microbiota and intestinal permeability, aggravating portal dyscrasia and generating detrimentalcycles between steatosis, inflammation, fibrosis, and concomitant vasoregulatory disturbances [65,66].

Hepatic vascular tone is determined by complex interactions of LSECs with other types of liver cells found in their vicinity, such as HSCs, Kupffer cells, and vascular smooth muscle cells [80]. LSECs possess endothelial nitric oxide synthase (eNOS), which is responsible for the production of nitric oxide (NO), a key vasodilator molecule regulating sinusoidal flow within the liver [81]. In response to biomechanical, inflammatory, and immune-mediated signals, LSECs undergo distinct anatomical changes, including a loss of their fenestration and the development of basal lamina, negatively affecting molecular transport between portal blood and hepatocytes [82]. Moreover, structural changes in endothelial dysfunction are accompanied by diminished eNOS activity and NO generation as a hallmark of LSEC injury [81]. Steatosis is rapidly associated with LSEC capillarization, indicating an almost immediate impact on sinusoidal vasoregulation [75,83]. LSECs and other liver cells increasingly release various vasoconstriction mediators such as endothelin-1, thromboxane A_2_, and leukotrienes [84,85]. HSCs, which are wrapped around the sinusoidal channels, gain contractility due to the scarcity of LSEC-derived NO and the loss of its tonic inhibitory effect [84,86]. HSCs are further activated by mediators such as platelet-derived growth factor, insulin-like growth factor, endothelin-1, and eicosanoid derivatives released in response to liver injury [87].

In association with these vasoregulatory changes, increased portal pressure becomes detectable in experimentally induced MASLD shortly after the onset of steatosis [75]. Several studies have confirmed the early rise of portal pressure in animal models of diet-induced MASLD in the presence of severe steatosis, but without steatohepatitis or fibrosis, altered sinusoidal vasoresponsiveness has been attributed to NO deficiency and/or vasoconstrictor excess [67,68,69].

The involvement of gut microbiota in these vasoregulatory changes was elegantly demonstrated in the experimental model of MASLD induced by a high-glucose/high-fat diet when FMT from control-fed mice prevented the increase in portal pressure [57]. FMT in this study also ameliorated endothelial dysfunction and insulin resistance through restoration of the hepatic Akt-dependent eNOS signaling pathway. In addition, FMT partially reversed the selective activation of intestinal FXR, which is regulated by bile acids and has been implicated in insulin resistance [57]. It remains to be seen whether these findings can be translated to human MASLD. Further metabolomic analysis will be needed to determine what specific derivatives of the host metabolism or other bioactive substances linked to gut microbiota may account for these vasoregulatory effects, either by impairing pathways of vasorelaxation in LSECs or sensitizing these cells to vasoconstrictive stimuli [17].

Hydrogen sulfide (H_2_S), a gaseous vasoactive mediator, has been recognized as a link between gut microbiota and portal circulation [88,89]. H_2_S may be synthesized by the gut microbiota and through endogenous metabolic pathways. Fecally excreted H_2_S is the product of cysteine-fermenting and sulfur-reducing gut bacteria such as *Desulfovibrio* and *Bilophila*, which use sulfate as a terminal electron acceptor [90,91]. Microbial H_2_S has mostly been associated with negative biological effects, including impairment of the gut barrier and intestinal inflammation [92]. By contrast, endogenous H_2_S produced in the liver by several enzymes, including cystathionine-γ-lyase (CSE), has potential beneficial effects in MASLD, including mitigation of endothelial dysfunction, oxidative stress, and apoptotic cell death through signaling pathways such as AMPK, mTOR, and the NLRP3 inflammasome [93,94]. Both FXR and GBPAR1 are known to upregulate the expression of CSE and other enzymes involved in the endogenous production of H_2_S [70,71]. Since FXR and GBPAR1 are targeted by bile acids that are subject to modifications by the gut microbiota, it is tempting to speculate that dysbiosis in MASLD is associated with reduced intrahepatic H_2_S release that may contribute to sinusoidal vasoconstriction and increased intrahepatic vascular resistance. However, most of the relevant work on H_2_S pathobiology utilized the experimental model of CCl_4_-induced cirrhosis, and it is, therefore, unclear if the findings are applicable to MASLD.

The essential amino acid tryptophan reaches the colon when ingested in excess and becomes metabolized to indole by various bacteria such as *E. coli*, *Lactobacilli*, and *Clostridium* [95]. Indole is further transformed by the gut microbiota into indole-3-acetate, indole-3-propionate, tryptamine, and other derivatives that may have a protective role in MASLD [51]. Indole-3-acetate alleviated steatosis, oxidative stress, and liver inflammation in mice with high-fat diet-induced MASLD [52]. In a similar experimental model, the administration of indole-3-acetate induced PGC1-α expression and recovered mitochondrial respiratory capacity in liver cells [53]. Additional work linked high-fat diet-induced dysbiosis to the depletion of indole-3-acetate and tryptamine, while replacement of these metabolites attenuated the liver inflammatory response and adverse changes in lipid metabolism through multiple molecular targets [72]. There is little known about the vasoregulatory effects of portal-derived tryptophan metabolites in the liver. Portal hypertension was reduced by the intracolonic administration of indole and indoxyl in the rat model of thioacetamide-induced cirrhosis [73]. At variance with these beneficial effects, indole-3-propionate activated HSCs in vitro along with the upregulation of fibrogenic marker genes and increased contractility [74]. Additional studies are needed to elucidate the impact of tryptophan derivatives on sinusoidal circulation in MASLD.

## 4. Indirect Impact of Gut Microbiota on Sinusoidal Hemodynamics in MASLD

Structural changes in liver sinusoids increasingly account for the development of portal hypertension with the progression of fibrosis in chronic liver disease. However, there are multiple lines of evidence that steatosis and steatohepatitis present a mechanical barrier to sinusoidal flow and contribute to the gradual rise in portal pressure before significant fibrosis develops [19]. Steatotic hepatocytes compress the sinusoidal space down to half of its original size [96], resulting in tortuous and narrow vascular channels and reduced blood flow [68]. Inflammatory cellular infiltration, hepatocellular ballooning, and interstitial swelling in steatohepatitis may further reduce the diameter of sinusoids and increase intrahepatic vascular resistance [97,98]. By contributing to the development of steatosis and steatohepatitis through a variety of molecular mechanisms, dysbiosis is a conceivable source of these mechanical impediments associated with increased intrahepatic vascular resistance in MASLD.

An important component of steatohepatitis is believed to stem from exposure of the liver to increased amounts of portal-derived antigens from nutrients and gut microbiota [23]. Gut-derived microbe-associated molecular patterns (MAMPs) are molecular structures of commensal and pathogenic microorganisms that include bacterial wall components such as lipopolysaccharide or endotoxin (LPS) from Gram-negative bacteria, lipoteichoic acid and peptidoglycan from Gram-positive bacteria, or bacterial and viral DNA [99]. MAMPs activate pattern recognition receptors (PRRs) located in the cell membrane, intracellular compartment membranes, and the cytoplasm, inducing downstream signaling pathways that regulate inflammation, protect from infection, and maintain a balance of host microecology [100]. Toll-like receptors (TLRs) and Nod-like receptors (NLRs) are the best-known PRRs and represent a major component of the innate immune system [101]. In the liver, TLRs are expressed by most liver cell types, including LSECs, which remove food-derived and bacterial antigens and mitigate innate immune responses within the liver [102]. LSECs perform this function by utilizing at least seven types of cell surface and intracellular TLRs [99]. NLRs, including the NLRP inflammasomes, are intracellular sensors of microbial and danger signals that are expressed in immune cells, LSECs, HSCs, and hepatocytes with a complex role in the innate immune response of the liver [103]. In addition, LSECs possess various scavenger receptors that assist in the engulfment and breakdown of exogenous substances of microorganismal origin or modified endogenous biomolecules [99].

There is substantial evidence that dysbiosis and increased gut permeability in MASLD are associated with increased activation of PRRs, inducing pro-inflammatory and profibrotic responses in the liver. Serum and liver LPS levels are higher in diet-induced and human MASH in comparison with controls [76]. The expression of TLR4 and TLR9 is higher in the liver of patients with MASH (but not if only steatosis is present), and diet-induced experimental MASH is less severe in mice made genetically deficient in TLR9 [77]. Liver tissue bacterial DNA profiles in patients with obesity and MASLD confirm an over-representation of Proteobacteria, and increased amounts of DNA of additional taxa have been detected in association with the severity of obesity [78]. Innate immune responses, including the activation of Kupffer cells and recruited macrophages, release inflammatory cytokines and chemokines, leading to hepatocellular stress, injury, and death. These events generate damage-associated molecular patterns (DAMPs) that are recognized by the inflammasomes and promote the activation of the NLRP3 and caspase-1 pathways [79]. Recent work demonstrated that the de novo microbial metabolite 2-oleoylglycerol (2-OG) activates HSCs and promotes the synthesis of extracellular matrix (ECM) proteins in a macrophage-dependent manner involving NF-κB and TGF-β signaling [45]. Elevated 2-OG levels have also been detected in the liver tissue obtained from a small cohort of obese patients with or without histologically confirmed steatohepatitis [45].

## 5. Portal Metabolomics: The Holy Grail to Understanding MASLD?

Metabolomic analysis of portal blood for the identification and characterization of macromolecules derived from nutrients or from processes related to gut microbiota is essential to understanding the gut–liver crosstalk in health and disease. Many metabolites delivered to the liver via the portal circulation are transformed through pathways of energy metabolism and detoxification, accounting for very different concentrations in the portal and hepatic vein [48]. Certain substances with low abundance but potent biological activities on liver metabolism, inflammation, fibrosis, or vasoregulation may not even be detectable in the systemic circulation due to the first-pass effect in the liver [31]. Due to its secluded anatomy, however, metabolomic investigation of the portal vascular territory has been restricted to experimental models [104,105] or instances in which the human portal vein and its tributaries become accessible during the placement of transjugular intrahepatic portosystemic shunt [61,106], bariatric surgical interventions, [62,107], or liver transplantation from healthy donors [108].

Within these limitations, several studies have provided important insights into portal metabolomics and illustrate the importance of this approach in the understanding of liver pathobiology. In earlier work, lipidomic analysis was simultaneously performed on systemic and portal blood collected at the time of bariatric surgery from women with severe obesity in the absence or presence of MASH [62]. While the portal phospholipidome was less affected by the presence of MASH compared to dramatic changes seen in the systemic circulation, levels of phosphatidylglycerols and phosphatidylethanolamines in the portal vein were significantly higher in MASH. Since these lipid constituents are mostly found in bacterial membranes as opposed to eukaryotic cell membranes that are predominantly composed of phosphatidylcholines, these findings reflect the contribution of changing gut microbiota [62]. Untargeted metabolomic profiling of mice given a single fast-food meal identified several gut microbe-derived metabolites that became more abundant in portal blood when compared to the chow control group [105]. Since the analysis was performed 4 h after a single gavage of fast-food meal, the findings indicate that diet can quickly reshape the metabolic activity of gut microbiota. Moreover, fast food-induced differences in the level of portal-derived metabolites were similar to those in control and antibiotics-treated mice, implying the role of gut microbiota in the enrichment of these metabolites in the portal circulation [105].

Recent research has shed new light on the potential role of microbially produced ethanol in MASLD. In two different cohorts of patients undergoing bariatric surgery, portal vein ethanol levels were found to be higher in patients with biopsy-proven MASLD [109]. In addition, postprandial levels of ethanol-induced by a standardized mixed meal (and measured after eliminating the hepatic first-pass effect by the selective inhibition of alcohol dehydrogenase) were significantly higher in individuals with MASH, while this effect was abolished following the administration of antibiotics to deplete gut microbiota. Differential abundance analysis in this study linked postprandial ethanol levels to *Streptococcus* and *Lactobacillus* species [109]. These provocative results underscore the limitations one may encounter when inferring hepatic exposure to portal metabolites from peripheral blood analysis. Specifically, the surprising findings that microbial capacity in MASLD may produce ethanol levels that exceed the legal driving limit blur the line between alcohol-related liver disease and MASLD and bestow new meaning on the novel disease entity of MetALD caused by a combination of MASLD and increased alcohol intake [1].

The emerging field of endohepatology, in particular EUS-guided vascular interventions, represents a potentially game-changing opportunity for the research of portal metabolomics and hemodynamics [34,110]. This technique, consisting of transgastric puncture of the portal and hepatic veins to directly measure the portal pressure gradient, was primarily developed to allow for a less invasive and more accurate alternative to the method of HVPG assessment, which has been almost exclusively used in cirrhosis. EUS-PPG can be combined with liver tissue sampling [111] and blood collection [112] from the portal and hepatic veins. A particularly promising aspect of EUS-guided portal sampling is that it can provide insight into the pre-cirrhotic stages of MASLD that have been essentially out of reach due to the invasive nature and inherent risks of HVPG. Using EUS-guided portal sampling may facilitate the identification of pre-hepatic metabolomic signatures associated with MASLD of different severity and correlate metabolite levels with increases in portal pressure [112]. While EUS is not without potential complications, it is less invasive and may become an acceptable diagnostic approach to the evaluation of patients with noncirrhotic MASLD.

## 6. Conclusions

MASLD originates in metabolic dysfunction and develops at the crossroads of anatomical structures and functional pathways. While the pathophysiology of MASLD remains incompletely understood, the gut–liver axis emerges as a critical framework for this process, with portal circulation as the primary connection. The immense and diverse population of gut microbiota has a myriad of ways to exert beneficial and harmful effects, involving biotransformation of nutrients, production of de novo metabolites, release of microbial structural components, and impairment of the intestinal barrier with impact on innate immunity, metabolism, inflammation, fibrosis, and vasoregulation in the liver and beyond. These mechanisms can heavily influence the onset and progression of MASLD while many specific details remain hidden due to the fact that access to the portal vein is limited. While we have a good understanding of clinically significant portal hypertension, knowledge in the field of portal pathobiology relevant to the early stages of chronic liver disease is still in its infancy. To address this major unmet need, future studies must focus on creating safe and reliable diagnostic tools to intercept portal metabolites in relation to gut microbiota, exploring their effect on liver health and progression of liver damage, allowing for the timely assessment and monitoring of hemodynamic and biochemical parameters within the portal circulation, and using this information to develop novel and targeted therapeutic interventions for all stages of MASLD.

## Data Availability

Not applicable.

## Abbreviations

2-OG, 2-oleoylglycerol; AMPK, AMP-activated protein kinase; CA, cholic acid; CCl_4_, carbon tetrachloride; CDCA, chenodeoxycholic acid; CSE, cystathionine-γ-lyase; CSPH, clinically significant portal hypertension; DAMP, damage-associated molecular pattern; DCA, deoxycholic acid; EC, endothelial cell; ECM, extracellular matrix; eNOS, endothelial nitric oxide synthase; ET-1, endothelin-1; EUS, endoscopic ultrasound; FMT, fecal microbiota transplantation; FXR, farnesoid X receptor; GPBAR1, G protein-coupled bile acid receptor; H_2_S, hydrogen sulfide; HSC, hepatic stellate cell; HVPG, hepatic venous pressure gradient; IC, immune cells; IPA, indole-3-propionate; KC, Kupffer cell; LCA, lithocholic acid; LPS, lipopolysaccharide; LSEC, liver sinusoidal endothelial cell; LTA, lipoteichoic acid; MAMP, microbe-associated molecular pattern; MASLD, metabolic dysfunction-associated steatotic liver disease; MASH, metabolic dysfunction-associated steatohepatitis; mTOR, mammalian target of rapamycin; NAFLD, nonalcoholic fatty liver disease; NASH, nonalcoholic steatohepatitis; NF-κB, nuclear factor kappa B; NLR, Nod-like receptors; NLRP, nucleotide-binding domain, leucine-rich–containing family, pyrin domain-containing; PA, phenylacetate; PE, phosphoethanolamines; PG, phosphoglycerols; PPG, portal pressure gradient; PRR, pattern recognition receptor; SCFA, short-chain fatty acid; SD, space of Disse; TGF-β, transforming growth factor-beta; TLR, Toll-like receptor; WHVP, wedged hepatic venous pressure.
